# Health Inequity of Stage and Survival of Gastric Cancer in California

**DOI:** 10.3390/cancers17223596

**Published:** 2025-11-07

**Authors:** Philip H. G. Ituarte, Kevin Sullivan, Marta M. Jankowska, Rebecca Nelson, Robert Huang, Matthew C. Hernandez, Chi Wan Wong, Supriya Deshpande, I. Benjamin Paz, Laleh Melstrom, Edward S. Kim, Yuman Fong, Yanghee Woo, on behalf of the Social Determinants of Health Research Working Group

**Affiliations:** 1 Department of Surgery, City of Hope National Medical Center, Duarte, CA 91010, USA; pituarte@coh.org (P.H.G.I.); kesullivan@coh.org (K.S.); matchernandez@salud.unm.edu (M.C.H.); sudeshpande@coh.org (S.D.); bpaz@coh.org (I.B.P.); lmelstrom@coh.org (L.M.); yfong@coh.org (Y.F.); 2 Department of Population Sciences, Beckman Research Institute, City of Hope National Medical Center, Duarte, CA 91010, USA; mjankowska@coh.org; 3 Department of Computational and Quantitative Medicine at the Beckman Research Institute, City of Hope National Medical Center, Duarte, CA 91010, USA; rnelson@coh.org; 4 Division of Gastroenterology and Hepatology, Department of Medicine, Stanford University, Stanford, CA 94305, USA; rjhuang@stanford.edu; 5 Department of AI and Data Science, Beckman Research Institute, City of Hope National Medical Center, Duarte, CA 91010, USA; alecwong@coh.org; 6 City of Hope Lennar Foundation Cancer Center, Irvine, CA 92618, USA; edwkim@coh.org

**Keywords:** gastric cancer, health disparities, ethnic minorities, comorbidities, early detection, Korean American, Hispanic American

## Abstract

**Background:** Gastric cancer (GC) remains a significant health burden in the U.S, particularly among ethnic minorities. We identified patient-level risk factors contributing to advanced-stage (AS) diagnosis and poor survival to guide strategies to address GC-related health disparities. **Methods:** We conducted a retrospective cohort analysis of 18,396 histologically confirmed GC cases (4102 early-stage (ES) and 14,294 AS) diagnosed between 2000 and 2019, using data from the California Cancer Registry linked to the California Office of Statewide Health Planning and Development. Eligible cases were adults age ≥ 18 with complete diagnostic and follow-up data. Multivariable logistic and Cox regression models were used to identify predictors of AS-GC and five-year disease-specific (DSS) and overall-survival (OS) outcomes. Analyses were further stratified by Asian and Hispanic subgroups. **Results:** Korean heritage was the strongest predictor of ES-GC [OR 0.58 (95% CI, 0.47–0.71), *p* < 0.001] and was independently associated with the lowest GC-specific mortality risk [HR 0.73 (95% CI: 0.67–0.80), *p* < 0.0001]. The youngest age group (18–44 years) had the highest AS-GC rate (91.4%). Asian ethnicity, receipt of care at NCI-designated cancer centers, and prior upper endoscopy were associated with improved OS and DSS. In contrast, comorbidities such as GERD, diabetes, liver disease, smoking and alcohol abuse, and older age ≥ 75, U.S.-birth, and rural residence were linked to worse outcomes. **Conclusions:** Distinct demographic, clinical, and healthcare access factors contribute to disparities in GC outcomes. These findings support the development of culturally tailored early-detection programs, and risk-based screening for GC care, particularly in vulnerable populations.

## Simple Summary

Gastric cancer (GC) disproportionately affects racial and ethnic minorities in the U.S. Although GC mortality has declined, disease burden remains unequally distributed, contributing to health inequities. In this largest ethnically enriched, population-based study of GC in the U.S., race, ethnicity, socioeconomic factors, and environmental factors jointly influenced stage at diagnosis and survival outcomes. Korean patients had the highest likelihood of early-stage diagnosis and the lowest GC-specific mortality, whereas Mexican, Filipino, and uninsured patients were more likely to present with advanced disease. Younger age (<45 years), male sex, and comorbidities such as GERD, smoking, and type II diabetes also were associated with worse outcomes and should be considered in identifying at-risk individuals. These findings support the development of race- and ethnicity-informed risk stratification tools and highlight the need for targeted early-detection strategies, including upper GI endoscopy, to reduce GC mortality and promote health equity in an increasingly diverse U.S. population.

## 1. Introduction

Gastric adenocarcinoma (GC) remains a major global health challenge and a leading cause of cancer-related mortality worldwide [1]. Although relatively uncommon in the U.S., GC imposes a disproportionate burden on racial and ethnic minority populations. Geographic and ethnic disparities in incidence, stage at diagnosis, and survival outcomes highlight significant inequities in early detection and access to care. For example, in countries like South Korea and Japan where there are high incidences of GC, national screening programs have led to early-stage (ES) diagnosis in over 60% of cases, resulting in markedly improved survival over the course of their successful implementation. In contrast, the majority of the GC cases in Western countries without screening programs, including the U.S., are detected at advanced stages (AS) and associated with significantly worse prognosis [1,2].

Importantly, the incidence and mortality of GC in the U.S. are unequally distributed by ethnicity. Korean, Japanese, and Hispanic Americans face significantly higher risks of developing GC compared to non-Hispanic Whites (NHWs) [3,4]. More specifically, Korean Americans exhibit the highest incidence, four to five times greater than that of NHWs as shown in prior analyses of the California Cancer Registry (CCR) data. While risk factors for GC such as *Helicobacter pylori* [5] or Epstein–Barr virus infections [6], age, male sex, tobacco use [7,8,9], dietary patterns low in fiber or high in salt [10,11,12,13,14,15,16], and obesity [17] are well-documented contributors to GC development, less is known about how these and other clinical and socioeconomic factors [18,19] influence stage at diagnosis and long-term survival in the U.S. [20,21].

Understanding how demographic, clinical, and socioeconomic factors influence GC presentation and outcomes is critical for identifying high-risk populations and guiding targeted early-detection strategies—especially in low-incidence countries where screening is not standard practice. To address this gap, we conducted a large, population-based study using linked data from the CCR and the California Office of Statewide Health Planning and Development (OSHPD). Our objective was to identify demographic, clinical, and healthcare access factors associated with AS diagnosis and poor survival in GC, with particular attention to racial and ethnic disparities in outcomes.

## 2. Methods

### 2.1. Patients

Gastric adenocarcinoma (GC) cases diagnosed between 2000 and 2019 were identified from the California Cancer Registry (CCR) using site and histology codes defined by the American Joint Committee on Cancer (AJCC) 6th, 7th, or 8th Edition. The inclusion criteria were histologically confirmed GC as the first or only primary malignancy, age ≥ 18 years, known AJCC stage at diagnosis, and complete follow-up data. Cases with in situ disease, missing tumor size, “zero tumor size”, or “no tumor found” were excluded. ES-GC was defined as AJCC stage I, and AS-GC as stages II–IV.

Each CCR case was linked at the patient level by patient identifier to hospital and ambulatory surgery discharge records from the OSHPD. The primary and secondary diagnoses and procedures codes were extracted in ICD-9-CM and ICD-10 formats. This study was approved by the Institutional Review Board (IRB #13479) and the California State Committee for the Protection of Human Subjects (#2019-225).

### 2.2. Variables and Definitions

Demographic, clinical, and treatment-related risk factors were extracted from variables available in CCR. Race/ethnicity was categorized into non-Hispanic White (NHW), non-Hispanic Black (NHB), Native American, Hispanic subgroups (Mexican, Cuban/Puerto Rican, Central/South American, other Hispanic), and disaggregated Asian/Pacific Islander subgroups (e.g., Korean, Japanese, Filipino, Chinese, Vietnamese, Cambodian/Thai, Laotian/Hmong, South Asian, Hawaiian/Samoan, other Asian). Socioeconomic status (SES) was measured at the census tract level based on a composite index of income, education, employment, and housing characteristics [22].

Additional variables included birthplace (U.S.-born, non-U.S.-born, unknown), tumor site, size, grade, and histology (intestinal, diffuse, or other epithelial type). Treatment variables included receipt of upper gastrointestinal endoscopy (UGIE), surgery at the primary site, and chemotherapy. Rural-Urban Commuting Area (RUCA) classification and whether there was treatment at a National Cancer Institute (NCI)-designated center were also studied.

Common co-existing medical conditions and comorbidities associated with GC risk were identified using ICD-9 and ICD-10 diagnosis codes (Table S1). These included *H. pylori*, peptic ulcer disease (PUD), gastroesophageal reflux disease (GERD), diabetes mellitus type II (DM-II), obesity, smoking, alcohol dependency, and presence of liver disease associated with cirrhosis. Measures of PUD and liver disease were based on ICD-9 or ICD-10 diagnosis codes associated with these subsets of chronic disease represented in the Deyo–Charlson comorbidity scale [23]. Metabolic syndrome was defined by a validated ICD-9-based algorithm and was included as a possible risk factor [24,25]. We translated ICD-9 codes into corresponding ICD-10 equivalent codes using a web-based crosswalk (Convert ICD-9-CM Codes to/from ICD-10-CM/PCS (accessed on 3 November 2025)). Comorbidity score was measured by applying the Deyo modification of the Charlson Comorbidity Index, excluding cancer-related subscales.

### 2.3. Statistics

For univariable analyses comparing ES-GC to AS-GC, Student’s *t*-test or chi-square were applied. Multivariable stepwise logistic regression with backward entry comparing ES-GC to AS-GC was performed. Stepwise entry was applied as there was no a priori assumption about the impact of potential risk factors on outcomes. For overall survival (OS) or disease-specific survival (DSS) (GC mortality) outcomes at five years, Cox proportional hazards analysis with backward stepwise entry was also applied. Residuals were examined to ensure that the proportional hazards assumption was met. The inclusion criterion in multivariable models was *p* < 0.20 for stepwise models, and *p* < 0.05 was used to identify significant predictors of AS-GC or survival time. All analyses were performed using Stata/MP version14.2 (Stata Corp, College Station, TX, USA).

## 3. Results

### 3.1. Univariable Analyses of Early Versus Advanced Stage Gastric Cancer

A total of 18,396 GC cases from 2000 to 2019 met our predetermined inclusion criteria. There were 22.3% ES-GC (stage I; n = 4102) and 77.7% AS-GC (stage II–IV; n = 14,294) cases. As expected, differences in patient, tumor, and treatment factors were observed between ES-GC and AS-GC (Table 1). We used raw percentages to compare ES-GC and AS-GC, and the highest percentage of AS-GC was observed in the youngest patient group (18–44; 91.4%), while the lowest was observed in patients aged 75 years and greater (68.2%). A greater percentage of men (78.2%) than women (77.0%) had AS-GC. For race/ethnicity, the highest percentage of AS-GC was observed in Mexicans (85.0%), followed by Filipinos (82.6%), Hawaiian or Samoans (84.9%), and Cambodian or Thai (82.4%). Low percentages of AS-GC were observed among Cuban or Puerto Ricans (70.8%), Other Pacific Islanders (70.0%), and Other Asian (67.6%), while the lowest percentage was observed in Koreans (59.8%). Very low (80.0%) or low SES (78.2%) were associated with a higher frequency of AS-GC compared with middle and higher SES. Uninsured (86.6%) and Medicaid (85.5%) cases were associated with a higher frequency of AS-GC compared with the lowest frequency for health insurance represented by Medicare (70.7%). Surprisingly, patients with a comorbidity score of zero had a higher percentage of AS-GC (79.4%) compared with those with a score of 2 or higher (71.9%). Patients treated at NCI cancer centers had a slightly lower frequency of AS-GC (76.1%) compared with those treated at non-NCI facilities (77.9%; *p* = 0.05). Poorly differentiated grade (85.6%), largest tumor size (>7 cm; 93.3%), stomach-overlapping sites (88.7%), and diffuse histopathology (84.0%) were significantly associated with AS-GC. Receipt of UGIE (69.2%) was associated with lower frequency of AS-GC compared to cases without UGIE (78.4%, *p* <0.001).

Frequency of ES-GC versus AS-GC did not differ significantly in patients with liver disease, alcohol abuse, or smoking compared to those without these medical conditions. Surprisingly, patients with chronic conditions like metabolic syndrome, peptic ulcer disease, *H. pylori*, GERD, diabetes type II, and obesity had lower frequency of AS-GC compared with those without these conditions (Table 1). Patients living in urban areas had a higher frequency of AS-GC (77.8%) compared with those living in relatively rural-large town areas (71.8%) or rural-isolated areas (75.9%).

### 3.2. Multivariable Analyses and Early- Versus Advanced-Stage Gastric Cancer

After stepwise procedure eliminated several variables, the resulting multivariable logistic regression model identified factors associated with AS-GC risk (Table 2). Patients aged 18–44 had a higher risk compared with the reference group (65–74-year-old), and women had a lower risk compared to men. Compared with NHW, only Koreans, Native Americans, South Asians, and Asian-other had significantly lower odds of AS-GC among all racial/ethnic groups. Middle SES, a comorbidity score of ≥2, antrum site, surgery, and large-rural town residence had lower odds of AS-GC. The area under the curve for this logistic regression model was 0.90, indicating a good fit between the data and the statistical model.

As expected, compared with well-differentiated tumor grade, moderately well-differentiated and poorly/undifferentiated grades were significantly associated with greater odds of AS-GC. Diffuse or intestinal pathology and chemotherapy had higher odds of AS-GC. Figure S1 shows a forest plot of odds ratios from this model.

### 3.3. Multivariable Overall Survival

Greater mortality risk included patients aged 75 or older (HR 1.25, 95% CI 1.19–1.30, *p* < 0.001), who had nearly 25% greater mortality risk than 65–74-year-olds. Being NHW (reference category) was presumed to be associated with relatively worse survival as all other race/ethnicity categories were under 1.0, except for Laotian or Hmong (HR 1.07; *p* = 0.549). U.S. birthplace was associated with a 9% higher risk (HR 1.09, 95% CI 1.04–1.13, *p* < 0.001), and NCI treatment was associated with a 10% lower mortality risk (HR 0.90, 95% CI 0.86–0.95, *p* < 0.001).

As expected, risk of death increased with higher stage and tumor grade. Antrum and lesser curvature of stomach were associated with lower risk, but overlapping sites were associated with higher risk (HR 1.08, 95% CI 1.04–1.21, *p* = 0.055). Surgery and chemotherapy treatments were associated with lower mortality risk. Positive LN status, liver disease, smoking, and diabetes were each associated with increased mortality risk. Surprisingly, obesity was associated with significantly lower mortality risk (HR 0.86, 95% CI 0.82–0.91, *p* < 0.001). Rural area (large rural town) was associated with greater mortality risk (Table 3). Figure S2 shows a forest plot summarizing the HRs from this model.

### 3.4. Multivariable Disease-Specific Survival

The multivariable Cox model for DSS (Table 3) indicated a greater mortality risk for patients aged 75 or older (HR 1.22, 95% CI 1.16–1.29, *p* < 0.001). Compared with NHW race/ethnicity, a lower risk was observed for the Mexican, Central/South American, Chinese, Japanese, Filipino, Vietnamese, Other Asian groups, while the Korean group (HR 0.73, 95% CI 0.67–0.80, *p* < 0.001) had the lowest mortality risk. Compared with private insurance, Medicare was associated with lower mortality risk, as was treatment at an NCI-designated center (HR 0.90, 95% CI 0.85–0.95, *p* < 0.001). Compared with non-U.S. births, patients born in the U.S. had an 8% greater mortality risk (HR 1.08, 95% CI 1.04–1.13, *p* < 0.001) (Table 3).

Overlapping sites within the stomach was associated with greater mortality risk (HR 1.12, 95% CI 1.03–1.22, *p* = 0.011), whereas lesser curvature of the stomach (HR 0.86, 95% CI 0.79–0.95, *p* < 0.002) was associated with lower risk compared with fundus, the reference group. Diffuse (HR 1.21, 95% CI 1.03–1.22, *p* < 0.001) but not intestinal histopathology was associated with greater mortality risk compared with other epithelial histology. Increasing levels of AJCC stage and tumor grade were associated with worse DSS. UGIE (HR 0.85, 95% CI 0.79–0.92, *p* < 0.001), surgery (HR 0.38, 95% CI 0.35–0.41, *p* < 0.001), and chemotherapy (HR 0.66, 95% CI 0.64–0.69, *p* < 0.001) again demonstrated lower risk. Positive lymph nodes (LNs) (HR 2.41, 95% CI 2.23–2.61, *p* < 0.001), liver disease, smoking, diabetes, and GERD were associated with greater DSS risk (Table 3). As with OS, obesity was associated with lower DSS risk (HR 0.86, 0.81–0.91, *p* < 0.001). Compared with urban areas, residence in rural-large towns was associated with greater risk (HR 1.21, 95% CI 1.07–1.36, *p* = 0.003). Figure S3 shows a forest plot summarizing HRs from this model.

## 4. Discussion

In this large, population-based cohort of over 18,000 GC patients, we found that more than three-quarters (77.7%) presented with AS disease (stage II–IV), consistent with known patterns of late GC diagnosis in the U.S. We identified multiple sociodemographic, clinical, and system-level factors independently associated with stage at presentation and survival, emphasizing persistent disparities and the multifactorial determinants of GC outcomes.

### 4.1. Disparities in Stage at Diagnosis

To our knowledge, this is the first population-based study of an ethnically enriched U.S. population to identify the impact of a comprehensive set of patient-specific factors on AS-GC diagnosis and five-year DSS and OS. AS-GC was more frequent among younger patients, men, and certain racial/ethnic minority groups including Mexicans, Filipinos, Hawaiians/Samoans, and Cambodians/Thais.

Socioeconomic disadvantages, public (e.g., Medicaid) or no insurance, and urban residence were also linked with higher odds of advanced disease. These findings highlight structural barriers that delay diagnosis, such as limited access to primary care, language and cultural obstacles, and delay in symptom recognition or endoscopic referrals.

Unexpectedly, patients without other medical conditions were more likely to present with advanced disease—suggesting that individuals with chronic conditions may have more frequent interactions with the healthcare system that lead to increased opportunities for detection. This is further supported by our finding that the receipt of UGIE was strongly associated with ES diagnosis, reinforcing its value in early detection. Notably, younger patients (18–44 years), a group typically perceived as low-risk, had the highest risk of AS-GC—likely due to both delayed presentation and low clinical suspicion. Previously, we had shown that young Hispanic men were most likely to be diagnosed with stage-IV GC with peritoneal disease [26]. As GC often remains asymptomatic in ES and younger patients are less likely to be concerned about GC, patients may present after many months of GC-associated symptoms. Moreover, lack of GC awareness may contribute to symptoms being overlooked or misdiagnosed within the medical communities, further delaying GC diagnosis. Potentially reflecting good baseline performance status and ability to tolerate more aggressive treatments, no difference was seen in this age group with respect to OS, compared to the reference age group (65–74 years).

We also found striking differences by ethnicity; NHW, NHB, and Hmong patients were at highest risk for AS-GC and had the poorest survival. Conversely, Korean patients were more likely to be diagnosed with ES-GC and had significantly better DSS and OS. This likely reflects a combination of cultural awareness, provider vigilance, and healthcare practices imported from South Korea, where national screening has improved early detection and outcomes [27]. In Korean American communities, especially in Los Angeles, endoscopic screening may be more frequently recommended and accepted, despite the absence of a formal U.S. screening program [28,29].

Interestingly, Central/South American patients had better DSS and OS than NHW. These findings are not readily explained by earlier stage of diagnosis, as multivariable analysis demonstrated no significant difference in ES-GC versus AS-GC diagnosis for these patients. A plausible explanation includes the so-called “Hispanic paradox,” referring to lower mortality risk for Hispanics compared to NHW or NHB patients for several health conditions [30]. However, studies are mixed with respect to this effect, with some showing improved mortality in Hispanic cancer patients [31,32,33,34] but not others [35,36]. Ultimately, the cause of improved OS or DSS in subgroups of Hispanic patients cannot be determined by this retrospective population-based registry study.

Surprisingly, the presence of comorbidities (excluding smoking and alcohol use) was associated with ES-GC, and patients with a history of *H. pylori* infection had improved survival. This likely reflects greater healthcare engagement and opportunities for symptom evaluation and endoscopy. While we could not directly assess endoscopy rates by subgroup, our findings support the hypothesis that healthcare access and utilization influence stage at diagnosis.

### 4.2. Pathologic and Treatment Predictors of Stage and Survival

As expected, poorly differentiated tumors, larger tumor size, diffuse histology, and non-antral location were strongly associated with AS disease. On multivariable survival analyses, increasing age, U.S. birthplace, higher tumor grade, diffuse histology, and nodal positivity were associated with worse OS and DSS. Conversely, treatment at NCI-designated centers, surgical resection, and chemotherapy were associated with significantly improved outcomes, underscoring the importance of guideline-based, multidisciplinary management.

The finding that U.S.-born patients had higher mortality compared with foreign-born individuals parallels prior studies, possibly reflecting differences in tumor biology, diet, *H. pylori* exposure, or health behaviors. Additionally, racial/ethnic groups such as Koreans, Central/South Americans, and Filipinos had lower GC mortality than NHW, even after adjustment, pointing to the influence of cultural, environmental, or genetic factors that warrant further exploration [37].

Interestingly, obesity was associated with lower GC mortality risk, which may reflect a form of the “obesity paradox” observed in other cancers, potentially due to better nutritional reserves, different tumor biology, or reverse causality where weight loss is an early symptom of aggressive disease.

### 4.3. Limitations and Implications

Our findings should be interpreted in the context of several limitations. While this study leveraged comprehensive registry data, limitations include incomplete capture of comorbidities, lifestyle factors, and treatment details. Also, socioeconomic and geographic measures were assigned based on census and residential data and may not fully reflect individual-level access barriers.

Despite these constraints, the findings highlight actionable disparities in GC diagnosis and survival. Expanding access to diagnostic endoscopy, increasing awareness, and implementing culturally tailored outreach strategies could improve outcomes, especially in ethnic subgroups with high burden and limited access to care.

## 5. Conclusions

In conclusion, this study demonstrates a multifactorial relationship among race, ethnicity, and socioeconomic/environmental factors. Identifying AS-GC risk factors can inform risk stratification for endoscopic screening efforts and community outreach in targeted high-risk populations to increase GC awareness. Our study suggests that younger age (<45 years), male sex, and comorbidities such as GERD, smoking history, and DM-II should be considered in identifying at-risk patients. Future work to disentangle the complex interplay among these factors can be aimed at evaluating the impact of other social determinants of health on survival and ethnicity in GC.

## Figures and Tables

**Table 1:** Frequencies for variables by early- versus advanced-stage gastric cancer (n = 18,358).

		ESG	ASG		
		Stage I	Stage II–IV	Total	
Variable	Category	n = 4102 (%)	n = 14,294 (%)	n = 18,396	*p*
Age, median (IQR)		72.5 (62–81)	65.0 (53–75)	67.0 (55–77)	<0.001
Age Group	18–44	156 (8.6)	1662 (91.4)	1818	<0.001
	45–54	406 (15.3)	2244 (84.7)	2650	
	55–64	668 (17.8)	3085 (82.2)	3753	
	65–74	1090 (24.0)	3460 (76.0)	4550	
	75+	1781 (31.7)	3841 (68.2)	5622	
Sex	Male	2206 (21.8)	7934 (78.2)	10,140	0.05
	Female	1896 (23.0)	6360 (77.0)	8256	
Race	non-Hispanic White	1042 (21.8)	3713 (78.2)	4755	<0.001
	non-Hispanic Black	284 (22.0)	1009 (78.0)	1293	
	Mexican	414 (15.0)	2340 (85.0)	2754	
	Cuban or Puerto Rican	14 (29.2)	34 (70.8)	48	
	Central/South American	180 (17.3)	863 (82.7)	1043	
	Hispanic other	701 (21.0)	2642 (79.0)	3343	
	Native American	20 (27.4)	53 (72.6)	73	
	Chinese	334 (25.1)	999 (74.9)	1333	
	Japanese	152 (28.4)	384 (71.6)	536	
	Filipino	89 (18.4)	394 (82.6)	483	
	Hawaiian or Samoan	11 (15.1)	62 (84.9)	73	
	Korean	463 (40.2)	688 (59.8)	1151	
	Vietnamese	146 (22.7)	497 (77.3)	643	
	Laotian or Hmong	21 (22.8)	71 (77.2)	92	
	Cambodian or Thai	13 (17.6)	61 (82.4)	74	
	South Asian	32 (25.2)	95 (74.8)	127	
	Other Pacific Islander	21 (30.0)	49 (70.0)	70	
	Other Asian	147 (32.5)	306 (67.6)	453	
	Other race/ethnicity	18 (34.6)	34 (65.4)	52	
Marital status	Not married	1740 (22.9)	5867 (77.1)	7607	0.115
	Married	2362 (21.9)	8427 (78.1)	10,789	
Socioeconomic status	Very low	690 (20.0)	2763 (80.0)	3453	0.003
	Low	739 (21.8)	2648 (78.2)	3387	
	Middle	694 (22.9)	2341 (77.1)	3035	
	High	634 (22.3)	2215 (77.7)	2849	
	Very high	600 (23.6)	1944 (76.4)	2544	
	Missing	745 (23.8)	2383 (76.2)	3128	
Insurance	Private/PPO/HMO	1313 (19.8)	5317 (80.2)	6630	<0.001
	Medicare	2042 (14.5)	4920 (70.7)	6962	
	Medicaid	424 (14.5)	2497 (85.5)	2921	
	Uninsured	118 (13.4)	759 (86.6)	877	
	Other	127 (20.1)	506 (79.9)	633	
	Missing	78 (20.9)	295 (79.1)	373	
Birthplace	Not USA	1586 (21.9)	5653 (78.1)	7239	0.303
	USA	756 (21.9)	2701 (78.1)	3457	
	Unknown	1760 (22.9)	5940 (77.1)	7700	
Comorbidity score	Zero	2020 (20.6)	7787 (79.4)	9807	<0.001
	One	1183 (21.9)	4207 (78.1)	5390	
	Two or more	899 (28.1)	2300 (71.9)	3199	
NCI cancer center	No	3582 (22.1)	12,642 (77.9)	16,224	0.05
	Yes	520 (23.9)	1652 (76.1)	2172	
Tumor grade	Well-differentiated	164 (64.6)	90 (35.4)	254	<0.001
	Moderately well	365 (28.1)	933 (71.9)	1298	
	Poorly/Undifferentiated	700 (14.4)	4165 (85.6)	4865	
	Missing	2873 (24.0)	9106 (76.0)	11,979	
Tumor size (cm)	>0–2	1325 (65.6)	695 (34.4)	2020	<0.001
	>2–3	592 (37.9)	968 (62.1)	1560	
	>3–5	605 (19.3)	2535 (80.7)	3140	
	>5–7	280 (13.8)	1744 (86.2)	2024	
	>7	156 (6.7)	2169 (93.3)	2325	
	Unknown	1144 (15.7)	6145 (84.3)	7289	
Tumor site	Fundus	194 (20.6)	749 (79.4)	943	<0.001
	Body	603 (22.9)	2025 (77.1)	2628	
	Antrum	1556 (28.2)	3959 (71.8)	5515	
	Pylorus	196 (24.5)	604 (75.5)	800	
	Lesser curvature	598 (27.3)	1592 (72.7)	2190	
	Greater curvature	247 (24.2)	773 (75.8)	1020	
	Overlapping sites	257 (11.3)	2018 (88.7)	2275	
	Unspecified	451 (14.9)	2574 (85.1)	3025	
Histopathology	Diffuse	1025 (16.0)	5381 (84.0)	6406	<0.001
	Intestinal	2682 (25.3)	7913 (74.7)	10,595	
	Other	385 (28.4)	973 (71.6)	1358	
Upper GI endoscopy	No	3680 (21.6)	13,348 (78.4)	17,028	<0.001
	Yes	422 (30.8)	946 (69.2)	1368	
Surgery	No surgery	1034 (11.9)	7635 (88.1)	8669	<0.001
	Surgery	3067 (31.5)	6657 (68.5)	9724	
Chemotherapy	No	3252 (36.7)	5603 (63.3)	8855	<0.001
	Yes	796 (8.7)	8366 (91.3)	9162	
	Unknown	54 (14.3)	325 (85.7)	379	
Positive lymph nodes	Negative	2437 (67.2)	1188 (32.8)	3625	<0.001
	Positive	235 (4.1)	5451 (95.9)	5686	
	Unknown	1430 (15.7)	7655 (84.3)	9085	
Liver disease	None	4049 (22.3)	14,138 (77.7)	18,187	0.36
	Mild	31 (27.9)	80 (72.1)	111	
	Severe	22 (22.5)	76 (77.5)	98	
Alcohol abuse	No	3996 (22.3)	13,941 (77.7)	17,937	0.678
	Yes	106 (23.1)	353 (76.9)	459	
Smoking	No	3732 (22.4)	12,933 (77.6)	16,665	0.332
	Yes	370 (21.4)	1361 (78.6)	1731	
Metabolic syndrome	No	3440 (21.3)	12,686 (78.7)	16126	<0.001
	Yes	662 (29.2)	1608 (70.8)	2270	
Peptic ulcer disease	No	3651 (21.8)	13,093 (78.2)	16,744	<0.001
	Yes	451 (27.3)	1201 (72.7)	1652	
*H. pylori*	No	3805 (22.0)	13,470 (78.0)	17,275	<0.001
	Yes	297 (26.5)	824 (73.5)	1121	
GERD	No	2971 (21.6)	10,777 (78.4)	13,748	<0.001
	Yes	1131 (24.3)	3517 (75.7)	4648	
DM II	No	3082 (20.8)	11,727 (79.2)	14,809	<0.001
	Yes	1020 (28.4)	2567 (71.6)	3587	
Obesity	No	3595 (22.0)	12,774 (78.9)	16,369	0.002
	Yes	507 (25.0)	1520 (75.0)	2027	
RUCA	Urban	3981 (22.2)	13,924 (77.8)	17,905	0.047
	Large rural area	82 (28.2)	209 (71.8)	291	
	Small/isolated rural	39 (24.1)	123 (75.9)	162	

**Table 2:** Multivariable logistic regression for early- versus advanced-stage cancer (n = 18,396).

Variables	Categories	OR (95% CI)	*p*-Value
Age group	18–44	2.39 (1.92–2.97)	<0.001
	45–54	1.23 (1.05–1.45)	0.013
	55–64	1.24 (1.07–1.44)	0.004
	65–74 (reference)		
	75+	0.73 (0.64–0.83)	<0.001
Sex	Male (reference)		
	Female	0.9 (0.81–0.99)	0.026
Race	non-Hispanic White (reference)		
	non-Hispanic Black	0.96 (0.78–1.18)	0.691
	Mexican	1.01 (0.85–1.20)	0.893
	Cuban or Puerto Rican	0.91 (0.37–2.20)	0.832
	Central or South American	0.92 (0.73–1.16)	0.500
	Hispanic other	0.88 (0.75–1.02)	0.083
	Native American	0.44 (0.22–0.87)	0.018
	Chinese	0.94 (0.76–1.15)	0.522
	Japanese	0.93 (0.69–1.25)	0.628
	Filipino	1.08 (0.78–1.50)	0.638
	Hawaiian or Samoan	1.57 (0.70–3.52)	0.272
	Korean	0.58 (0.47–0.71)	<0.001
	Vietnamese	0.90 (0.68–1.18)	0.431
	Laotian or Hmong	0.74 (0.42–1.29)	0.281
	Cambodian or Thai	1.64 (0.72–3.72)	0.235
	South Asian	0.48 (0.29–0.80)	0.005
	Other Pacific Islander	0.53 (0.25–1.13)	0.099
	Other Asian	0.7 (0.52–0.94)	0.018
	Other race/ethnicity	0.37 (0.19–0.72)	0.003
Comorbidity	None (reference)		
	One	1.00 (0.89–1.12)	0.970
	Two or more	0.82 (0.71–0.94)	0.004
Socioeconomic status	Very low (reference)		
	Low	1.02 (0.87–1.19)	0.841
	Middle	0.85 (0.73–1.00)	0.056
	High	1.05 (0.88–1.24)	0.608
	Very high	0.97 (0.81–1.16)	0.747
	Missing	0.99 (0.84–1.18)	0.949
NCI cancer center	No (reference)		
	Yes	0.89 (0.76–1.03)	0.116
Tumor grade	Well-differentiated (reference)		
	Moderately well	1.98 (1.26–3.13)	0.003
	Poorly/Undifferentiated	2.37 (1.54–3.66)	<0.001
	Unknown	1.41 (0.92–2.15)	0.111
Tumor size	>0–2 (reference)		
	>2–3	2.35 (1.93–2.85)	<0.001
	>3–5	4.97 (4.16–5.94)	<0.001
	>5–7	7.91 (6.41–9.77)	<0.001
	>7 cm	13.54 (10.61–17.27)	<0.001
	Unknown	3.72 (3.17–4.36)	<0.001
Tumor site	Fundus (reference)		
	Body	0.90 (0.70–1.14)	0.375
	Antrum	0.74 (0.59–0.92)	0.008
	Pylorus	0.92 (0.67–1.27)	0.617
	Lesser curvature	0.78 (0.61–1.01)	0.057
	Greater curvature	0.86 (0.63–1.15)	0.305
	Overlapping site	1.19 (0.92–1.56)	0.188
	Unspecified	1.25 (0.98–1.60)	0.078
Histopathology	Other epithelial (reference)		
	Diffuse	1.66 (1.37–2.01)	<0.001
	Intestinal	1.49 (1.25–1.78)	<0.001
Upper GI endoscopy	No (reference)		
	Yes	0.90 (0.75–1.08)	0.255
Surgery	No (reference)		
	Yes	0.15 (0.13–0.18)	<0.001
	Unknown	0.41 (0.07–2.38)	0.320
Chemotherapy	No (reference)		
	Yes	3.69 (3.29–4.14)	<0.001
	Unknown	2.35 (1.55–3.54)	<0.001
Lymph node status	Negative (reference)		
	Positive	34.59 (29.28–40.86)	<0.001
	Unknown	1.91 (1.59–2.30)	<0.001
Metabolic syndrome	No (reference)		
	Yes	0.87 (0.75–1.01)	0.071
*H. pylori*	No (reference)		
	Yes	0.86 (0.70–1.06)	0.151
RUCA	Urban (reference)		
	Large rural	0.65 (0.45–0.95)	0.025
	Small rural/isolated	0.78 (0.49–1.26)	0.311

**Table 3:** Multivariable models for overall and disease-specific survival at 5 years (n = 24,212).

		Overall Survival		Disease-Specific Survival	
Variables	Categories	HR (95% CI)	*p*-Value	HR (95% CI)	*p*-Value
Age group	18–44	0.95 (0.90–1.01)	0.13	0.95 (0.88–1.02)	0.135
	45–54	0.97 (0.92–1.03)	0.324	0.98 (0.92–1.04)	0.470
	55–64	0.96 (0.91–1.01)	0.104	0.95 (0.90–1.01)	0.088
	65–74 (reference)				
	75+	1.25 (1.19–1.30)	<0.001	1.22 (1.16–1.29)	<0.001
Race	non-Hispanic White (reference)				
	non-Hispanic Black	1.04 (0.98–1.11)	0.183	1.03 (0.96–1.10)	0.393
	Mexican	0.97 (0.92–1.02)	0.284	0.93 (0.88–0.99)	0.016
	Cuban or Puerto Rican	0.87 (0.70–1.08)	0.219	0.84 (0.64–1.12)	0.233
	Central or South American	0.86 (0.79–0.92)	<0.001	0.82 (0.75–0.90)	<0.001
	Hispanic other	0.98 (0.94–1.03)	0.44	0.97 (0.92–1.02)	0.183
	Native American	0.87 (0.65–1.17)	0.36	0.85 (0.63–1.15)	0.285
	Chinese	0.81 (0.75–0.86)	<0.001	0.79 (0.73–0.85)	<0.001
	Japanese	0.81 (0.74–0.9)	<0.001	0.83 (0.75–0.92)	0.001
	Filipino	0.91 (0.82–1.01)	0.070	0.82 (0.73–0.92)	0.001
	Hawaiian or Samoan	1.02 (0.84–1.25)	0.843	0.78 (0.60–1.02)	0.071
	Korean	0.70 (0.65–0.76)	<0.001	0.73 (0.67–0.80)	<0.001
	Vietnamese	0.80 (0.73–0.88)	<0.001	0.84 (0.75–0.93)	0.001
	Laotian or Hmong	1.07 (0.85–1.35)	0.549	1.09 (0.84–1.41)	0.504
	Cambodian or Thai	0.81 (0.62–1.06)	0.130	0.77 (0.57–1.05)	0.103
	South Asian	0.87 (0.72–1.04)	0.128	0.81 (0.64–1.02)	0.072
	Other Pacific Islander	0.93 (0.68–1.26)	0.628	0.80 (0.56–1.12)	0.195
	Other Asian	0.86 (0.77–0.97)	0.015	0.87 (0.76–0.99)	0.040
	Other race/ethnicity	0.50 (0.37–0.68)	<0.001	0.44 (0.30–0.63)	<0.001
Marital status ^a^	Unmarried (reference)				
	Married	0.97 (0.94–1.01)	0.119		
Insurance ^b^	Private/PPO/HMO (reference)				
	Medicare			0.91 (0.87–0.96)	<0.001
	Medi-Cal			0.99 (0.94–1.05)	0.743
	Uninsured			0.98 (0.89–1.08)	0.649
	Other			0.95 (0.87–1.05)	0.321
	Missing			0.95 (0.86–1.07)	0.409
Birthplace	Not USA				
	USA	1.09 (1.04–1.13)	<0.001	1.08 (1.04–1.13)	<0.001
Comorbidity score ^c^	Zero (reference)				
	One			0.96 (0.93–1.00)	0.081
	Two+			1.01 (0.95–1.07)	0.757
NCI Cancer Center	No				
	Yes	0.90 (0.86–0.95)	<0.001	0.90 (0.85–0.95)	<0.001
Stage	I (reference)				
	II	1.39 (1.29–1.51)	<0.001	1.52 (1.38–1.67)	<0.001
	III	2.05 (1.90–2.21)	<0.001	2.39 (2.18–2.61)	<0.001
	IV	3.07 (2.88–3.28)	<0.001	3.58 (3.31–3.89)	<0.001
	Unknown	1.83 (1.72–1.95)	<0.001	2.15 (1.99–2.32)	<0.001
Tumor size	>0–2 (reference)				
	>2–3	1.21 (1.11–1.32)	<0.001	1.37 (1.24–1.52)	<0.001
	>3–5	1.44 (1.34–1.55)	<0.001	1.62 (1.48–1.77)	<0.001
	>5–7	1.58 (1.46–1.71)	<0.001	1.83 (1.67–2.01)	<0.001
	>7 cm	1.81 (1.68–1.95)	<0.001	2.15 (1.96–2.36)	<0.001
	Unknown	1.57 (1.47–1.68)	<0.001	1.77 (1.63–1.93)	<0.001
Tumor grade	Well-differentiated (reference)				
	Moderately well	1.29 (1.06–1.58)	0.010	1.46 (1.13–1.89)	0.004
	Poorly/Undifferentiated	1.54 (1.27–1.85)	<0.001	1.75 (1.37–2.24)	<0.001
	Unknown	1.70 (1.41–2.05)	<0.001	2.01 (1.57–2.57)	<0.001
Tumor site	Fundus (reference)				
	Body	0.93 (0.86–1.00)	0.052	0.96 (0.88–1.04)	0.331
	Antrum	0.91 (0.85–0.98)	0.012	0.94 (0.87–1.02)	0.139
	Pylorus	0.95 (0.86–1.05)	0.297	0.95 (0.85–1.07)	0.421
	Lesser curvature	0.85 (0.79–0.93)	<0.001	0.86 (0.79–0.95)	0.002
	Greater curvature	0.93 (0.85–1.03)	0.164	0.94 (0.85–1.05)	0.284
	Overlapping site	1.08 (1.00–1.17)	0.055	1.12 (1.03–1.22)	0.011
	Unspecified	1.07 (0.99–1.15)	0.094	1.07 (0.98–1.16)	0.148
Histopathology	Other epithelial (reference)				
	Diffuse	1.18 (1.11–1.26)	<0.001	1.21 (1.13–1.31)	<0.001
	Intestinal	0.99 (0.93–1.06)	0.822	1.00 (0.93–1.07)	0.991
Upper GI endoscopy	No (reference)				
	Yes	0.88 (0.83–0.94)	<0.001	0.85 (0.79–0.92)	<0.001
Surgery	No (reference)				
	Yes	0.40 (0.38–0.44)	<0.001	0.38 (0.35–0.41)	<0.001
	Unknown	0.89 (0.50–1.59)	0.699	0.86 (0.45–1.62)	0.637
Chemotherapy	No (reference)				
	Yes	0.63 (0.61–0.65)	<0.001	0.66 (0.64–0.69)	<0.001
	Unknown	1.00 (0.91–1.10)	0.976	1.02 (0.92–1.14)	0.657
Lymph node status	Negative (reference)				
	Positive	2.14 (2.00–2.28)	<0.001	2.41 (2.23–2.61)	<0.001
	Unknown	1.97 (1.81–2.14)	<0.001	2.09 (1.89–2.31)	<0.001
Liver disease	None				
	Mild	1.31 (1.08–1.59)	0.005	1.30 (1.04–1.61)	0.018
	Severe	1.37 (1.11–1.70)	0.003	1.28 (1.01–1.64)	0.045
Alcohol abuse ^d^	No				
	Yes	1.10 (0.99–1.23)	0.069		
Smoking	No				
	Yes	1.11 (1.06–1.18)	<0.001	1.07 (1.01–1.14)	0.027
DM II	No				
	Yes	1.15 (1.10–1.20)	<0.001	1.14 (1.08–1.19)	<0.001
GERD	No				
	Yes	1.05 (1.02–1.09)	0.006	1.07 (1.03–1.12)	0.001
*H. pylori*	No				
	Yes	0.95 (0.89–1.01)	0.127	0.96 (0.90–1.04)	0.322
Obesity	No				
	Yes	0.86 (0.82–0.91)	<0.001	0.86 (0.81–0.91)	<0.001
RUCA	Urban (reference)				
	Large rural	1.18 (1.06–1.31)	0.003	1.21 (1.07–1.36)	0.003
	Small rural/isolated	1.12 (0.96–1.30)	0.142	1.13 (0.96–1.32)	0.15

^a^ Only in OS model. ^b^ Only in DSS model. ^c^ Only in DSS model. ^d^ Only in OS model.

## Data Availability

Y.W. had full access to all the data in the study and takes responsibility for the integrity of the data and the accuracy of the data analysis. The original contributions presented in this study are included in the article/Supplementary Materials. Further inquiries can be directed to the corresponding author.

## Supplementary Materials

The following supporting information can be downloaded at https://www.mdpi.com/article/10.3390/cancers17223596/s1, Figure S1: Forest plot of logistic regression of ES-GC versus AS-GC; Figure S2: Forest plot of Cox proportional hazard model for 5-year overall survival; Figure S3: Forest plot of Cox proportional hazard model for 5-year disease-specific survival; Table S1: Common co-existing medical conditions and those associated with GC risk captured using ICD-9 and ICD-10 diagnosis codes.
