# Supplementary material for: Health Inequity of Stage and Survival of Gastric Cancer in California
Source: Cancers (Basel). 2025 Nov 7;17(22):3596. doi: 10.3390/cancers17223596 (PMC12651519; doi:10.3390/cancers17223596)
Supplement: Supplementary file 1 [file cancers-17-03596-s001.zip › cancers-3958179-supplementary.pdf]

cancers

MDPI

Supplementary Materials: Health Inequity of Stage and Survival of Gastric Cancer in California

Philip H. G. Ituarte, Kevin Sullivan, Marta M. Jankowska, Rebecca Nelson, Robert Huang, Matthew C. Hernandez, Chi Wan Wong, Supriya Deshpande, I Benjamin Paz, Laleh Melstrom, Edward S. Kim, Yuman Fong, Yanghee Woo and on behalf of the Social Determinants of Health Research Working Group

Table S1. Common co-existing medical conditions and those associated with GC risk captured using ICD-9 and ICD-10 diagnosis codes.

Diagnosis	Description	ICD-9 code	ICD-10 codes
H. pylori	Helicobacter pylori, or gastric ulcer due to H. pylori	041.86	B96.81
GERD	Reflux esophagitis	530.11	K21.0
GERD	Gastroesophageal reflux disease (GERD)	530.81	K21.9
GERD	Personal history of GERD	V12.79	Z87.19
Peptic ulcer disease	Chronic or unspecified peptic ulcer	531.40 - 531.71; 532.40 - 532.71; 533.40 - 533.71; 534.40 - 534.71;	K25.0; K25.4 - K25.7; K26.4 - K26.7; K27.4 - K27.7; K28.4 - K28.7; K28.9
Obesity	Obesity unspecified	278.00	E66.9
Obesity	Morbid obesity	278.01	E66.01
Obesity	Overweight	278.02	E66.3
Obesity	Obesity hypoventilation	278.03	E66.2
Obesity	History of bariatric surgery status	V45.86	Z98.84
Alcoholism	Acute alcoholic intoxication	303.00 - 303.03	F10.229
Alcoholism	Other & unspecified alcoholic dependence	303.90 - 303.93	F10.20, F10.21
Liver disease	Mild liver disease	571.2, 571.5, 571.6; 571.40-570.49	K70.30; K73.0 - K73.9; K75.4, K74.0; K74.60 - K74.69; K74.3 - K74.5
Liver disease	Severe liver disease	572.2, 572.3, 572.4, 572.8, 456.0, 456.1, 456.20, 456.21	K72.10 - K72.91; K76.6 - K76.7; I85.00 - I85.11
Smoking	Tobacco use disorder, current or past history, smoking include. Nicotine dependence (ICD-10)	305.1	F17.200
Diabetes mellitus, type II		250.00, 250.02, 250.10,250.12, 250.20, 250.22, 250.30, 250.32, 250.40, 250.42, 250.50, 250.52, 250.60, 250.62, 250.70, 250.72, 250.80, 250.82, 250.90, 250.92	E11.00, E11.01, E11.09, E11.51, E11.641, E11.65, E11.69
Metabolic syndrome	Metabolic syndrome x	277.7	E88.81
Metabolic syndrome	BMI 30+ (obesity)	278.01, 278.03; V85.30 - V85.39; V85.40 - V85.49	E66.01, E66.2; Z68.30 - Z68.39; Z68.40 - Z68.45
Metabolic syndrome	Low HDL cholesterol; lipoprotein deficiencies	272.5, 272.6	E78.6, E88.1
Metabolic syndrome	High triglycerides; hyperlipidemia	272.1- 272.4	E78.1 - E78.4; E78.5
Metabolic syndrome	High blood pressure	401.0, 401.1, 401.9, 402.0, 402.1, 402.9, 403.00, 403.01, 403.90, 403.91, 404.00, 404.01, 404.02, 404.03,	I10, I16.9, I12.9, I12.0, I13.0, I13.10, I13.11, I13.2, I15.0, I15.8

		404.10, 404.11, 404.12, 404.13, 404.90, 404.91, 404.92, 404.93, 405.01, 405.09, 405.11, 405.19, 405.91, 405.99	
Metabolic syndrome	High fasting glucose	250.00, 250.02, 2510.10, 250.12, 250.20, 250.22, 250.30, 250.32, 250.40, 250.42, 250.50, 250.52, 250.60, 250.62, 250.70, 250.72, 250.80, 250.82, 250.90, 250.92	E11.00, E11.01, E11.10, E11.11, E11.29, E11.311, E11.319, E11.36, E11.39, E11.40, E11.51, E11.618, E11.620, E11.621, E11.622, E11.628, E11.630, E11.638, E11.641, E11,649, E11.65, E11.69, E11.8, E11.9, E13.10

ICD: International classification of diseases; H. pylori: Helicobacter pylori; GERD: Gastroesophageal reflux disease; BMI: Body mass index; HDL: high-density lipoprotein.

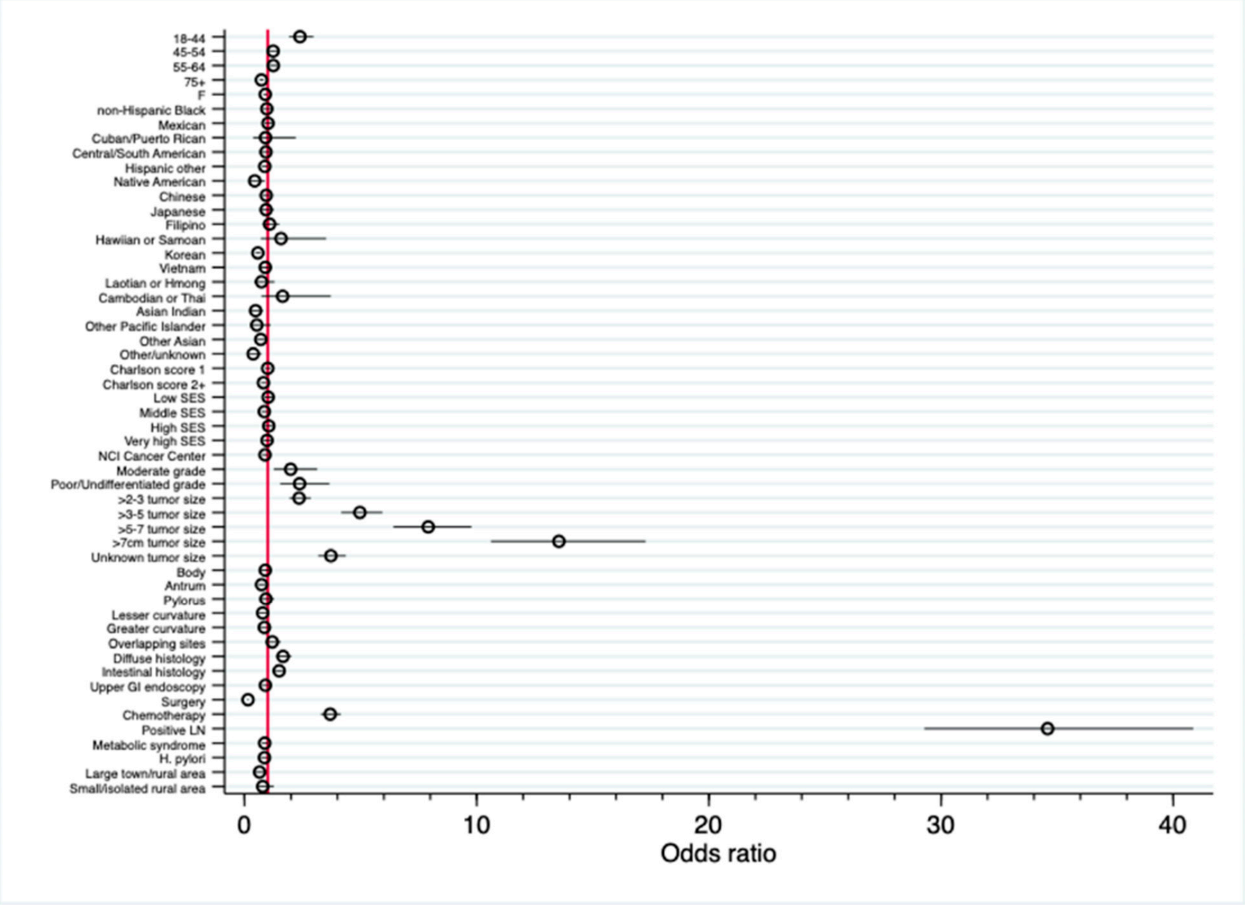

Figure S1. Forest plot of logistic regression of ES-GC versus AS-GC.

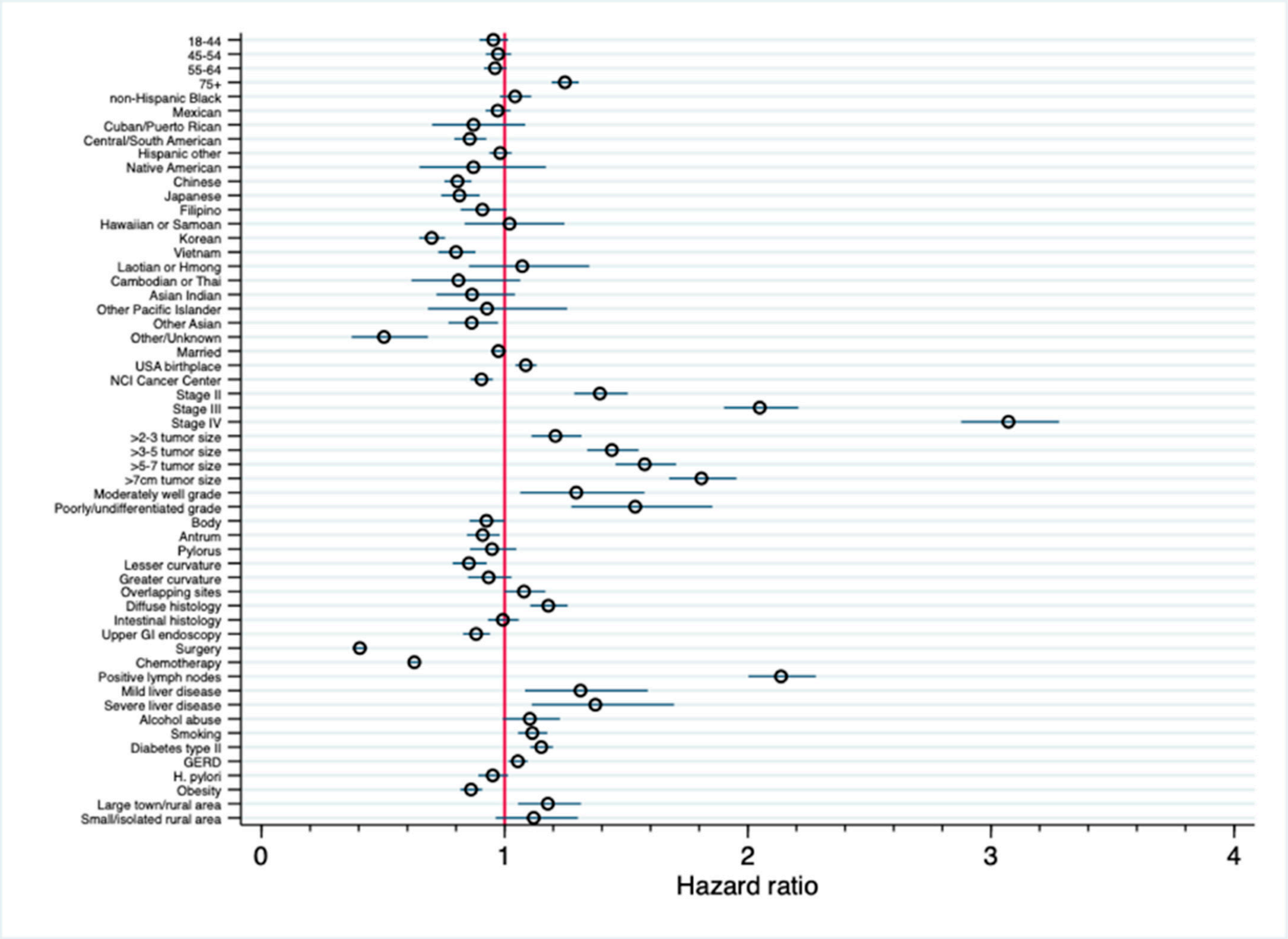

Figure S2. Forest plot of Cox proportional hazard model for 5-year overall survival.

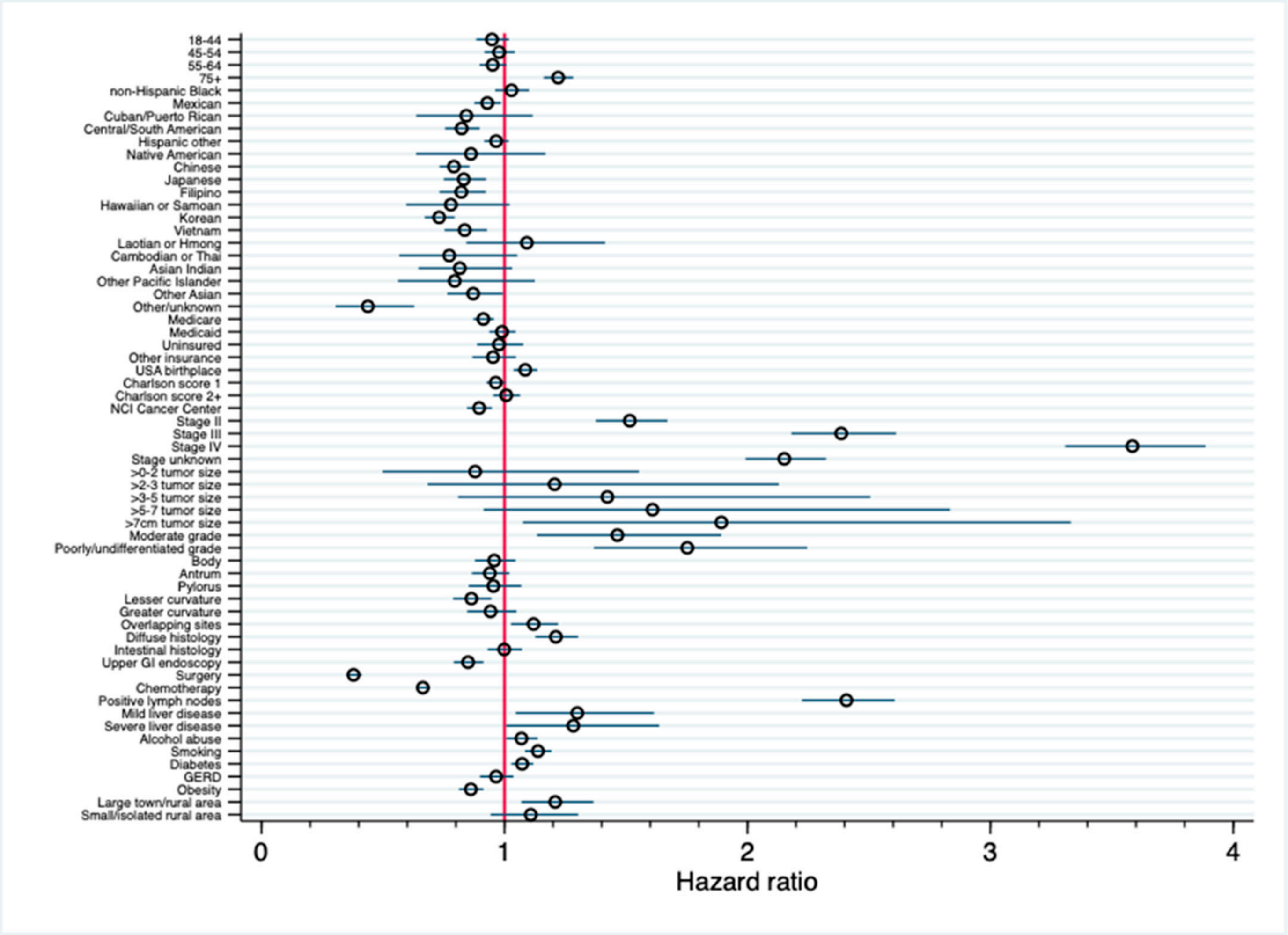

Figure S3. Forest plot of Cox proportional hazard model for 5-year disease-specific survival.
